# Supplementary material for: Mapping the transcriptional regulatory network of a fungal pathogen by exploiting transcription factor perturbation
Source: mBio. 2025 Nov 28;17(1):e02797-25. doi: 10.1128/mbio.02797-25 (PMC12802168; doi:10.1128/mbio.02797-25)
Supplement: Supplemental File Details — Details of supplemental files. [file mbio.02797-25-s0009.docx]

Supplementary files

**File_S1_TFs.xlsx**

A list of the 120 TFs that were deleted, the number of RNA-Seq replicates we have for each, and the 163 TFs that were used in the construction of the TF network map.

**File_S2_capsule_genes.tsv**

A list of 164 genes from FungiDB whose deletions have abnormal capsule phenotypes.

**File_S3_expr_reg_log2_fc_163_120.xlsx**

Differential expression of TF-encoding genes (Log_2_ fold change of TF deletion over wild-type). Each column provides data for a different TF deletion strain.

**File_S4_gene_go_association.txt**

GO annotations downloaded from the UniProt website and processed into a GAF file for input to the package GO-Term-Finder.

**File_S5_top_outdegree_top_indegree.xlsx**

Top 16 (10%) TFs that have the highest number of targets, and top 410 (10%) genes that have the highest number of regulators.

**File_S6_net_tf_tf.xlsx**

TF-TF pairs that have a Jaccard similarity greater than zero. The file includes the number of shared targets, Jaccard similarity index, p-value, and adjusted p-value.

**File_S7_tf_tf_pairs_mappings.xlsx**

1. (TF*_cn_*, TF*_sc_*) pairs that were mapped using the list of Kelliher et al. (Kelliher, et al., 2016)
2. (TF*_cn_*, TF*_sc_*) pairs that were mapped using homologous targets of *C. neoformans* and *S. cerevisiae*. If a Cryptococcus TF does not map to the S. cerevisiae TF at all, the columns “% identity”, “evalue”, and “qcov” are left blank.

qcov (query coverage) is the percentage of the query sequence that is covered by a BLASTP alignment with a sequence from the database.

**File_S8_tfs_not_mapped.xlsx**

Cryptococcus TFs that were not mapped to an *S. cerevisiae* TF by either our method or the Kelliher method. The file includes for each TF: the systematic name, the common name, whether it is known to regulate capsule, and the corrected p-value for enrichment with target genes that do not have *S. cerevisiae* homologs according to Kelliher et al. (Kelliher, et al., 2016).

Large files on Zenodo

The TF network map constructed using NetProphet3 cross-trained and integration modes.

<https://doi.org/10.5281/zenodo.17193243>

Differential expression of non-TF encoding genes (Log_2_ fold change of TF deletion over wild-type). Each column provides data for a different TF deletion strain.

<https://doi.org/10.5281/zenodo.17193388>

The DE network that was used to run NetProphet3. Rows are TFs and columns are target genes.

<https://doi.org/10.5281/zenodo.17193620>

The *S. cerevisiae* TF network map that was generated using NP3 10-fold cross validation (Abid and Brent, 2023) and used in this study. Format is tab separated values (.tsv).

<https://doi.org/10.5281/zenodo.17196637>
